# Anxiety, Depression, and Quality of Life in Women with Breast Cancer and Type 2 Diabetes: A Pilot Study in North Greece

**DOI:** 10.3390/jpm14121154

**Published:** 2024-12-17

**Authors:** Maria Parpori, Ioannis Tsamesidis, Evangelos Karamitrousis, Aikaterini Giakidou, Evangelia Kroustalidou, Polixeni Liamopoulou, Maria Lavdaniti

**Affiliations:** 1 Nursing Department, International Hellenic University, 57001 Thessaloniki, Greece; dbt712020@nurse.ihu.gr (M.P.);; 2 Department of Prosthodontics, School of Dentistry, Faculty of Health Sciences, Aristotle University of Thessaloniki, 54124 Thessaloniki, Greece; itsamesidis@auth.gr; 3 University Medical Oncology Department, Aristotle University of Thessaloniki, Papageorgiou General Hospital, 56429 Thessaloniki, Greece; karamitrousis@ihu.gr; 4 Section of “Agios Panteleimon”, Social Welfare Centre of Central Macedonia, 56430 Thessaloniki, Greece; 5 Surgical Department, University General Hospital of Alexandroupolis, 68131 Alexandroupolis, Greece; litsakroustalidou@gmail.com

**Keywords:** diabetes, type 2 diabetes, breast cancer, quality of life, anxiety, depression

## Abstract

**Introduction**: The incidence of breast cancer, as well as diabetes mellitus (DM), has continuously increased in recent years. The concurrent study of these diseases is particularly important, as there is a strong correlation between them due to hormonal, biochemical, and environmental factors. Moreover, the underlying metabolic dysfunction in this case could affect the treatment of breast cancer, as well as overall survival. In addition, the relationship of these two diseases with depression is not well studied. Patients with DM and cancer patients both experience depressive symptoms that have an impact on their mental health, as well as their quality of life. Personalized medicine offers a potential solution to these challenges by tailoring treatments to individual patient profiles. The present study will attempt to fill a gap in the existing literature regarding the relationship of patients with concurrent breast cancer and DM experiencing depression. More specifically, it will attempt to answer the question of whether there is a strong correlation between breast cancer, DM, and mental health in patients from a large geographical division of the country. **Methodology**: This was a cross-sectional study. A total of 120 female patients participated in the research, 60 with type 2 diabetes mellitus (T2DM) (group B) and 60 with T2DM and breast cancer (group A). The DASS-21 questionnaire was used to determine their levels of anxiety and depression, and ADDQoL-19 was used to measure the patients’ quality of life. **Results**: Regarding quality of life, women with T2DM and breast cancer showed a better quality of life (QoL) than women with T2DM. Women who were retired (*p* = 0.025) and consequently postmenopausal (*p* = 0.035) demonstrated the highest levels of QoL, while stage III cancer patients had lower ADDQoL-19 scores. Regarding mental health, in the women from group A, a positive correlation of treatment with the occurrence of anxiety and depression (*p* = 0.034) and a negative correlation of cancer stage with mental health was observed. The women in group B (those with T2DM only) showed better mental health outcomes related to their treatment (*p* = 0.009). However, both married and unmarried women in this study experienced mental health burdens, with no significant difference between the two groups in terms of mental health impact. These findings suggest that marital status did not significantly influence the mental health of the participants in this study. **Conclusions**: Women with T2DM and breast cancer generally presented a better clinical picture than women with T2DM alone. Therefore, the comorbidity of these two diseases did not seem to negatively affect the quality of life and mental health of these women.

## 1. Introduction

Type 2 diabetes (T2DM) and breast cancer are two prevalent chronic conditions that significantly affect millions of women’s quality of life worldwide. Both diseases pose considerable challenges to physical health and well-being, often necessitating complex treatment regimens and lifestyle modifications. It is well known that diabetes mellitus has been a chronic disease since ancient times and is due to a lack of insulin being produced by the β-cells of the islets of Langerhans of the pancreas [1,2]. Furthermore, diabetes is one of the most common and fastest-growing diseases worldwide, predicted to affect 693 million adults by 2045, at a >50% increase from 2017 [3].

The co-occurrence of type 2 diabetes mellitus (T2DM) and breast cancer presents a unique clinical challenge, as these two chronic conditions are often interlinked through shared risk factors, such as age, obesity, and hormonal imbalances, and can complicate each other’s management and impact on quality of life [4].

There is a strong correlation between the development of breast cancer and diabetes mellitus, and this is due to various hormonal factors, such as hyperglycemia, hyperinsulinemia, insulin growth factor secretion (IGFS), and several others. Furthermore, these patients have a greater risk of developing more aggressive forms of the disease, such as triple-negative breast cancer (TNBC), in which hormone receptors or human epidermal growth factor receptor (HER-2) is not expressed [5].

Both diseases are linked to lifestyle factors such as obesity, sedentary behavior, and poor dietary habits, as well as biological mechanisms, like hormonal imbalances and chronic inflammation. When T2DM and breast cancer co-occur, they can complicate one another’s treatment and exacerbate health risks. For instance, diabetes can increase susceptibility to certain types of breast cancer and worsen cancer prognosis, while breast cancer treatments, including chemotherapy and hormonal therapies, can disrupt glucose regulation and elevate the risk of diabetes progression [6].

The co-management of these conditions requires careful balancing, as the side effects of the treatments for one illness may complicate the other. Moreover, this dual diagnosis heightens the risk of psychological distress, including depression, further complicating treatment adherence and impacting quality of life [7]. Studying T2DM and breast cancer together is essential to understanding the full spectrum of the interactions between these diseases, optimize the therapeutic strategies, and improve the outcomes for individuals managing both conditions. By addressing this intersection, healthcare providers can develop more integrated care models to address the multifaceted needs of this growing patient population [8].

Moreover, women diagnosed with breast cancer are more vulnerable to negative mental health consequences, including the symptoms of anxiety and depression [9]. There is also a relationship between the diagnosis of breast cancer and the development of depressive symptoms in a large proportion of patients. This percentage is even higher in patients who have undergone mastectomy compared to patients who have undergone more conservative operations [10]. The majority of the literature on the psychological effects of breast cancer in young women is currently accessible and concentrates on the treatment of psychosocial concerns and the survivability of patients in the early stages of the disease. Unfortunately, there are not many studies investigating comorbidities such as type 2 diabetes in women in this context. A few studies in the literature, such as Storey et al. [11], have noted that women with breast cancer and diabetes are at high risk of a poor quality of life. In detail, their study revealed that women with both breast cancer and diabetes presented disturbed sleep, low attention function, low physical function, and fatigue. The study by Imran et al. [12] also showed that anxiety disorders have direct effects on a compromised quality of life, including global health status, different social domains, and patient symptoms. Focusing on the disease with targeted treatments while neglecting systemic metabolic dysfunction may lead to treatment failure and resistance to cancer therapy. As our knowledge of the pathways linking systemic metabolism to cancer expands, it will be easier to identify patients who will benefit from treatments for particular metabolic abnormalities or customize therapies in order to handle particular cancer targets that have been altered by metabolic dysfunction [13].

In order to address the complex interplay between type 2 diabetes (T2DM) and breast cancer, a promising approach is offered by personalized medicine, tailoring the treatment to the specific needs and characteristics of individual patients. By considering a person’s genetic makeup, lifestyle, and the molecular profile of both diabetes and cancer, personalized medicine aims to optimize the therapeutic outcomes while minimizing its side effects. For women with both conditions, personalized treatment regimens can address their unique challenges, such as managing insulin resistance and glycemic control during cancer treatment. This approach is crucial because T2DM and breast cancer often share common biological pathways, such as insulin resistance, hormonal influences, and inflammatory processes, that can affect treatment outcomes and disease progression [14]. In breast cancer patients with T2DM, personalized medicine can optimize the treatment efficacy and minimize adverse effects. For instance, medications such as metformin, a common diabetes drug, have shown potential benefits in some breast cancer cases due to its effects on glucose regulation and cancer cell metabolism [8]. Genetic testing can guide the choice of therapies further by identifying which patients may respond to specific treatments better, such as certain chemotherapy agents or targeted therapies, while considering the impact of these therapies on glucose control [15]. Additionally, personalized dietary and lifestyle interventions, informed by metabolic profiling, can be used to address obesity and insulin resistance, which are risk factors for both diseases and can influence the disease outcomes [16]. By integrating these customized strategies, healthcare providers can improve the quality of life and overall prognosis for women managing both breast cancer and T2DM. Personalized medicine not only enhances the efficacy of treatments but also supports the comprehensive management of these patients, addressing both cancer progression and diabetes management in a holistic and patient-centered manner. This study addresses the psychosocial challenges faced by women living with type 2 diabetes mellitus (T2DM) and breast cancer in Northern Greece, focusing on understanding their unique needs to inform future healthcare practices and support services. Given the lack of existing research in this area, our investigation seeks to explore the prevalence of anxiety and depression among women diagnosed with both conditions while also examining the differences in the quality of life between those with T2DM and breast cancer and those with T2DM only. Additionally, this study aims to identify potential correlations between anxiety, depression, and quality of life, as well as to evaluate how demographic factors (e.g., age, socioeconomic status) and clinical variables (e.g., disease stage, treatment regimen) influence these psychosocial outcomes. By addressing these gaps in the international literature, this research seeks to mobilize healthcare authorities toward targeted interventions that improve mental health outcomes and ensure a more comprehensive approach to managing these complex conditions.

## 2. Materials and Methods

The present study is a cross-sectional study. The sample consists of women with breast cancer and type 2 diabetes.

### 2.1. Sample

**Figure 1:**
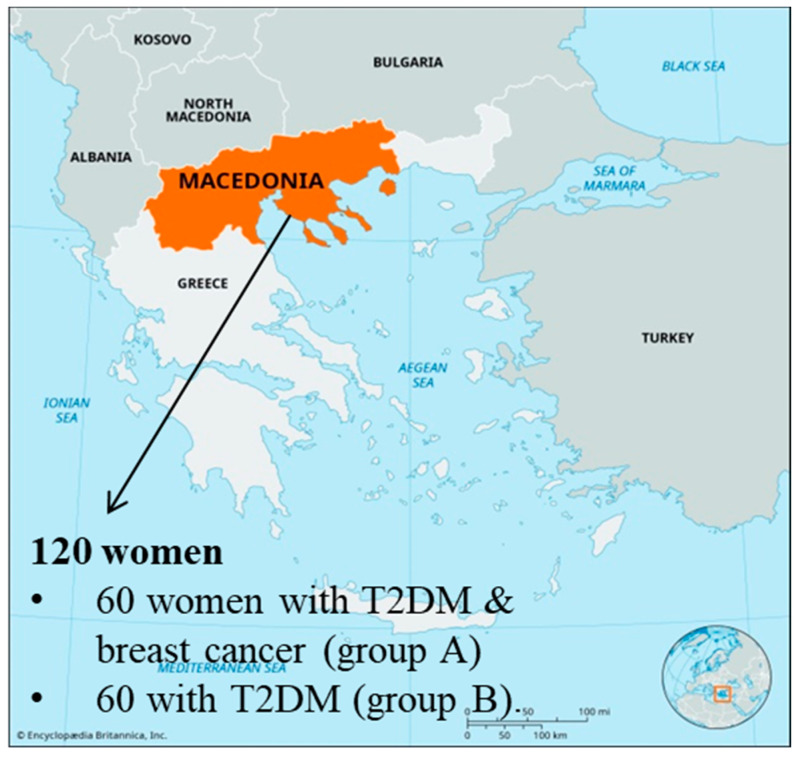
Map of the Macedonia region in Greece, highlighting the locations where the study sample was collected.

This study used a convenience sampling method, selecting a total of 120 women, divided into 60 women with both T2DM and breast cancer (group A) and 60 women with T2DM only (group B) from the region of Macedonia in Greece (Figure 1). Convenience sampling was chosen to allow for recruitment of accessible participants who met the entry criteria, given the challenges of identifying individuals with both conditions. The sample inclusion criteria for both groups were as follows: (a) Women aged 18 or older, regardless of education level, professional, or family status. (b) A duration of illness equal to or longer than six months. (c) Knowledge of the Greek language and writing ability. The sample characteristics considered included Body Mass Index (BMI), HbA1c, blood glucose levels, diabetes-related complications, cancer stage, and previous treatments. (d) The absence of severe psychiatric conditions or cognitive impairments that could interfere with self-reporting and comprehension of the study. (e) No other major comorbidities that could independently influence quality of life aside from the targeted diagnoses. (f) Specific cancer criteria for group A, which included women with a diagnosis of breast cancer in a stable phase or under active treatment, to ensure that cancer status was compatible with participation in the study. (g) Voluntary consent to participate after being informed of the study’s purpose and procedures. While this convenience sample may limit this study’s generalizability to some extent, the demographic and clinical profiles align with characteristics typical of the study population, offering valuable insights into quality of life and mental health in this unique patient group.

### 2.2. Measurement Tools

A questionnaire was used as the measurement tool in this study. It consisted of three parts, of which the first referred to the demographic characteristics of the patients, such as age; marital, occupational, and insurance status; educational level; and smoking and alcohol consumption, as well as characteristics related to type 2 diabetes, such as HbA1c levels and sugar levels, weight, height, and BMI; treatment for DM and any complications; hypertension; stage of cancer; previous treatment; and types of chemotherapy drugs. The second part was the DASS-21, which consists of 21 questions about depression, anxiety, and stress and is a 4-point Likert scale (from 0—Does not apply to me at all—to 3—Applies to me very much or most of the time). The anxiety scale assesses anxiety as a state, its stimulation of the autonomic nervous system, and its effect on the musculoskeletal system, while the depression scale assesses lack of interest, discomfort, devaluation of life, despair, and apathy. The scale to which each item belongs is indicated by the letters D for depression, A for anxiety, and S for stress. For each scale, the scores for the identified items are summed. Because the DASS-21 is a short version of the DASS, the final score for each item group (depression, anxiety, and stress) must be multiplied by two (×2). After being multiplied by 2, each score can be transferred to the DASS profile sheet, allowing for comparisons to be made between the three scales and also giving percentage scores. Higher scores for both the total scale and the subscales express higher levels of mental health burden. The DASS-21 interprets scores as follows: 0–14 indicates normal, 15–18 mild, 19–25 moderate, 26–33 severe, and 34–42 extremely severe stress levels (Table 1). The DASS-21 is generally considered a reliable and valid tool for assessing depression, anxiety, and stress in various populations. Its reliability is supported by strong internal consistency (Cronbach’s alpha, ranging from 0.80 to 0.90, which indicates good internal consistency) and good test–retest reliability [9].

The third part of the questionnaire was the Diabetes-Dependent Quality of Life (ADDQoL) check. ADDQoL consists of two survey parts: one measures general overall quality of life, and another 19 items are used to address the impact of diabetes on specific aspects of life. The 19 life domains are leisure activities; working life; journeys; holidays, physical health; family life; friendships and social life; close personal relationships; sex life; physical appearance; self-confidence; motivation to achieve things; people’s reactions; feelings about the future; financial situation; living conditions; dependence on others; freedom to eat; and freedom to drink. For these 19 domains, respondents are asked to rate what their lives would be like if they did not have diabetes. The scales range from −3 to +1 for the 19 life domains (the impact score) and from 0 to +3 for attributed importance (the importance score). A weighted score for each domain is calculated as a multiplier of the impact score and the importance score (ranging from −9 to +3). Lower scores reflect a poorer quality of life. Finally, a weighted average impact score (the ADDQoL score) is calculated for the entire scale across all of the applicable domains. ADDQoL-19 demonstrates good internal consistency, with its Cronbach’s alpha values typically between 0.70 and 0.90. It also exhibits strong test–retest reliability, with correlation coefficients of 0.80 to 0.90, meaning it can consistently measure quality of life in individuals with diabetes over time. The scale is a reliable and valid tool for assessing diabetes-related quality of life across a variety of populations [17].

### 2.3. The Data Collection Process

The questionnaire was distributed in printed form to women in the “Theagenio” Anticancer Hospital of Thessaloniki, “Papageorgiou” General Hospital of Thessaloniki, “University Hospital of Alexandroupolis”, and the Social Welfare Centre of Central Macedonia, section of “Agios Panteleimonas”. In detail, the printed questionnaires were distributed after the participants’ medical visits, where they were provided by the attending doctor. Participants were invited to fill out the questionnaire immediately after their consultation, with the support of healthcare staff if needed. This study was conducted in accordance with the Declaration of Helsinki and approved by the scientific councils (ethics committees) of the hospitals ((“Theagenio” Anticancer Hospital of Thessaloniki (Prot. No. 6531/14-4-2022; date of approval: 19 April 2022), “Papageorgiou” General Hospital of Thessaloniki (Prot. No. Δ3β/51106; date of approval: 25 October 2022), “University Hospital of Alexandroupolis” (Prot. No. 28466/24-06-2022; date of approval: 05 July 2022), and the Social Welfare Centre of Central Macedonia, section of “Agios Panteleimonas (Prot. No. 4516/2362022; date of approval: 14 July 2022)). Informed consent was obtained from all of the subjects involved in this study, with the patients stating that they agreed to take part to this research anonymously. The women’s participation in the research was voluntary, while 2 groups were created, women with T2DM and breast cancer (group A) and women with T2DM only (group B). The average time taken to complete the questionnaire was 10 min. Finally, each of the questionnaires obtained the necessary approval and valid translation. More specifically, the DASS-21 has been officially translated by Lyrakos and colleagues [13], and ADDQoL has been officially translated by Health Psychology Research, the UK.

### 2.4. Data Analysis

The data were initially collected in Excel sheets, and the subsequent statistical analysis was performed using IBM SPSS Statistics v23.0. For the descriptive statistics, continuous variables were expressed as means with standard deviations. The normality of the variables was assessed using the Kolmogorov–Smirnov test. For comparisons between independent groups, a parametric *t*-test was used to assess the differences in the means. The Pearson’s r correlation test was employed to examine the degree of correlation between variables, with the assumption that the data met the following criteria: normality, linearity, and homoscedasticity. The level of significance for all statistical tests was set at *p* < 0.05. No post hoc corrections were applied, as there were no multiple comparisons requiring such adjustments.

## 3. Results

### 3.1. Demographic Characteristics of Both Groups

A total of 120 female patients participated in the research, 60 with T2DM and breast cancer (group A) and 60 with T2DM (group B). Table 2 presents all of the demographic characteristics of the analyzed groups. The majority of the women who participated in the present study belonged to an age range of 46–60 years (49.5%), with a slight variation from the 61–85 age range (40.5%), which correlates with the fact that they were mostly retired (40.5%). Their main place of residence was cities (58.9%), and most of them had only finished primary school (38.5%).

### 3.2. Clinical Characteristics of Both Groups

Table 3 presents the clinical characteristics of all of the patients and each patient group separately. As shown in Table 2, the largest proportion (55.1%) of the women had been diagnosed with T2DM 1–10 years ago, and their main treatment was antidiabetic tablets (65.5%). Smoking and alcohol occurred in very low proportions in the women studied (24.1% (group A) and 8.65% (group B), respectively), and most of them did not report any diabetic complications. Their HbA1c levels were mostly <7 (76.7%), and most of the women were in the postmenopausal period (74%), without hypertension (53%). In detail, comparing the two groups, regarding the timeline of diagnosis, no significant differences were observed for the period of diagnosis (56.7% and 53.6% for group A and B, respectively). On the other hand, in terms of the treatment for T2DM in both groups of women, antidiabetic tablets were used as the main treatment more in group A (87% and 44% for groups A and B, respectively). However, there were also patients who had been diagnosed with T2DM more than 20 years ago, and it appeared that the patients with T2DM alone had more treatment options, with the next highest proportions being insulin (26%) and a combination of tablets and insulin (23%). As can be seen from Table 2, both groups of patients had low rates of smoking and alcohol consumption. According to their responses, usually, any patient who did not smoke did not consume alcohol either, while there were few patients who smoked without consuming alcohol. Regarding the diabetic complications that the patients may have had, the highest percentage in both categories was general complications (6.6% in group A and 43.5 in group B). In group A, the number of responses was the same for all complications, while in group B, the most common diabetic complications were vasculopathy and infarction, at 13%, and retinopathy and coronary artery disease, at 8.7%. Both groups were mostly postmenopausal, with a higher percentage for group A (87%), while this was 61% for group B. Regarding the clinical characteristics of cancer for group B, the highest percentage of patients were in cancer stage II (53%), while the fewest patients (7%) were found in stage I. Their main treatment was surgery (73%), while the minority of them followed radiotherapy (7%). Finally, all of the patients were on hormone therapy.

### 3.3. Quality of Life (ADDQoL)

Table 4, Table 5 and Table 6 present the results of the ADDQoL analysis for the quality of life of the two study groups. More specifically, Table 4 shows the correlation between the demographic characteristics presented in the first part of the results and the level of quality of life of the women in groups A and B, as ascertained using the ADDQoL questionnaire. Regarding group A, the correlations between gender, marital status, place of residence, occupation, insurance status, BMI, and smoking showed that only occupation affected quality of life (r = 0.456; *p* = 0.025). More specifically, the highest score (least negative) was obtained for retired women (mean: −0.57) and the lowest for female civil servants. None of the other correlation results were found to be statistically significant (n/s). Regarding group B, a significant impact on the quality of life of these participants was exerted by age and insurance status. Participants who were older than 61 years of age showed the highest score (less negative), indicating better quality of life. In terms of health insurance, it appeared that the women with private insurance experienced the worst quality of life.

Then, Table 5 shows the correlation of the clinical characteristics of the women in groups A and B with their quality of life. The correlations made with time of onset and T2DM treatment did not show statistically significant results. However, cancer stage always showed a negative correlation, with r = −0.550 (*p*-value = 0.034), with the quality of life of the women in the cancer group. Participants who were in stage III cancer had lower scores (more negative). In addition, blood glucose, hypertension, and menopause did not show any statistically significant trends. Regarding the correlations of the clinical characteristics of group B, a statistically significant association of menopause with these women’s quality of life was observed. More specifically, women in the postmenopausal period of their life showed higher scores (less negative score), and this was in agreement with the above result from Table 3 (a statistically significant correlation for women aged 61+).

Table 6 shows comparisons of the results (mean score) for each part of the ADDQoL questionnaire of the women in both groups. More specifically, regarding the general picture of their quality of life, the women in group A had better scores compared to the women in group B (*p* < 0.05). Regarding the key questions of what their life would be like if they did not have T2DM and the impact in terms of its significance, the group A women appeared to be more optimistic. Differences were found in the questions regarding their work and sex life, as well as their freedom to eat. In particular, the women in group A showed better scores and therefore a better quality of life than the women in group B, with a statistically significant difference (*p* < 0.05).

### 3.4. Anxiety and Depression (DASS-21)

The following, Table 7, Table 8, Table 9 and Table 10, present the results of the DASS-21 analysis to investigate the mental health of the two study groups. Table 7 presents the association between the demographic characteristics presented in the first part of the results and the mental health of the women in groups A and B, as ascertained from the DASS-21 questionnaire. Depression was negatively associated with occupation (r = −0.578; *p* = 0.003), while no other statistically significant association was found with age, marital status, educational level, place of residence, insurance status, or BMI. The correlations of their clinical characteristics with their DASS-21 scale scores indicated treatment of T2DM and cancer stage as key points in terms of the percentages of their influence on mental health in group A (Table 7). The treatment for type 2 diabetes mellitus (T2DM) was positively associated with better mental health outcomes in the women (*p* = 0.037), while a more advanced cancer stage was negatively associated with their mental health (*p* = 0.034). Additionally, insurance status was found to have a significant positive correlation with both depression and anxiety (r = −0.278, *p* = 0.036). However, no statistically significant correlations were identified with other factors, including age, marital status, educational level, place of residence, BMI, and smoking. Regarding clinical characteristics, treatment was positively associated with good mental health (r = 0.482; *p* = 0.009). The remaining correlations showed no statistically significant trends.

## 4. Discussion

The aim of this study was to investigate the existence of anxiety and depression in women with T2DM but also with breast cancer, as well as whether their level of mental health affects their quality of life. It is clear that comorbidity in chronic health problems negatively affects the quality of life of patients, as well as their mental health. In our study, specific influencing factors emerged from the demographic and clinical characteristics, which showed a trend in their impact on the patients’ quality of life and mental health.

More specifically, as emerged from the ADDQoL questionnaire, patients with T2DM and breast cancer who were retired and at an early stage of the disease had a better quality of life than all of the others, regardless of their occupation or stage of cancer. One possible explanation for this result is that individuals confronted with multiple serious diagnoses may develop heightened psychological resilience. Literature [18] suggests that life-threatening illnesses, such as cancer, often prompt individuals to re-evaluate their life priorities and adopt adaptive coping mechanisms, which can lead to a more positive outlook. This “post-traumatic growth” often results in a heightened appreciation for life, greater emotional stability, and better psychological adaptation. Consequently, these patients may place higher value on their current QoL, appreciating small improvements or finding meaning despite their health challenges.

The worst quality of life was presented by the women working as civil servants and in later cancer stages (III and IV). It is clear that an advanced stage of the disease is associated with a worse prognosis and is therefore a factor that increases patients’ anxiety.

Furthermore, in our research, depression was negatively associated with career choice. Patients with depression seem to have thought disorders and an inability to fulfill their goals; therefore, they have difficulty in seeking career opportunities.

On the other hand, the correlations of the clinical characteristics with the DASS-21 scale score results highlighted the medication of T2DM and cancer stage as key points in the rate of their influence on mental health. T2DM medication was positively associated with the patients’ mental health, while cancer stage was negatively associated. These results are in agreement with those of various studies, such as the systematic review by Alzahrani et al. [19]. Similarly, cancer stage was negatively associated with the patients’ poor quality of life, due to a shorter life expectancy in this case.

A study from Saudi Arabia [20] showed that emotional functioning is an important aspect which is directly correlated with patients’ feelings of satisfaction in palliative care and their daily routine, such as occupation. Another study [12] showed that anxiety disorders demonstrate a trend of compromised quality of life according to several scales, such as QLQ-CR30 and QLQ-BR23, including global health status, different social domains, and patients’ symptoms.

In patients with T2DM only, it seems that age and insurance status were significantly associated with a reduction in the level of their quality of life, in relation to the control group. A worse quality of life was observed in younger patients and in those with private insurance. Similar studies [21] have shown lower rates of quality of life in patients with a history of T2DM of more than 10 years and the presence of complications from the disease. Our research adds new factors to the existing literature. In particular, participants who were older than 61 years old (postmenopausal) showed the highest scores, highlighting their better quality of life, as well as quite low indicators of anxiety and depression. This finding aligns with the study by Gonzalez et al. [22], which noted that older women often exhibit better coping mechanisms, likely due to their accumulated life experience compared to their younger counterparts. Additionally, studies have shown that postmenopausal women tend to experience improved mental health after a cancer diagnosis, as they may perceive the diagnosis as a life-altering event that refocuses their attention to what is most meaningful [23]. Indeed, the control group of women with T2DM only presented poorer QoL and mental health scores compared to those with both T2DM and breast cancer. This counterintuitive finding might reflect the synergistic effect of managing multiple chronic diseases, which could lead individuals to develop a heightened sense of gratitude or resilience, improving their overall outlook despite the challenges [24]. The greater optimism observed in the women with both T2DM and breast cancer may also suggest that living with dual diagnoses promotes stronger psychological coping mechanisms, allowing these individuals to focus on improving their QoL rather than being overwhelmed by their conditions.

In terms of clinical characteristics, medication was positively associated with good mental health in the diabetic patients. Our finding is in agreement with those of previous studies [25] but also more recent ones [26]. The patients in the control group, surprisingly, presented lower scores in terms of their quality of life and mental health. Regarding the basic questions, the answers of the women with both diseases were more optimistic. The main differences were identified in the questions concerning their work and sex lives, as well as their free choice of diet. The women with T2DM and breast cancer, in all of the answers, showed a better quality of life and mental health. This finding is in agreement with a study in three racial groups that characterized sexual dysfunction as a negative factor in the quality of life of the study sample [27]. Sexual dysfunction, as noted in our study, emerged as a notable factor negatively affecting QoL, particularly among the women with T2DM. This is consistent with findings by Maiorino et al. [28], who identified sexual health as a critical but often overlooked domain in assessing QoL among diabetic patients. Furthermore, the impact of sexual dysfunction extends beyond physical intimacy, influencing self-esteem, relationship satisfaction, and overall mental health. Addressing these issues through integrated care models that include sexual health counseling could significantly enhance the QoL of affected individuals.

In terms of dietary choices, our findings reflect a tension between the restrictive dietary recommendations in T2DM management and the desire for personal freedom in food selection. Another study [29] highlights how rigid dietary constraints can exacerbate feelings of deprivation, leading to a diminished QoL. This suggests that patient-centered dietary interventions that incorporate individual preferences while maintaining glycemic control could improve satisfaction and mental well-being.

While this study identified associations between T2DM and breast cancer, it is important to note that causality could not be established due to the cross-sectional design. This limitation is consistent with other research on chronic diseases that relies on cross-sectional data, where longitudinal studies are needed to uncover the causal pathways and temporal dynamics. Future research employing a prospective cohort design could provide a deeper understanding of how these conditions influence each other over time, as demonstrated by studies that track long-term disease progression and treatment outcomes [30].

This study’s sample size and composition also posed challenges. Recruiting a sufficient number of patients with both T2DM and breast cancer was inherently difficult due to the rarity of this dual pathology and the impact of cancer-related mortality on diabetes outcomes. Similar challenges are commonly encountered in multimorbidity research, particularly when investigating rare disease combinations. For instance, studies on diabetes and cardiovascular disease often rely on national registries or multicenter collaborations to achieve the sample sizes necessary for robust analyses [31,32]. Adopting such multicenter approaches in future research could facilitate larger sample sizes and more comprehensive subgroup analyses.

Despite these limitations, this study provides valuable insights into the complex interplay between chronic conditions like T2DM and breast cancer. Addressing the challenges outlined above in future research will not only enhance the reliability and validity of our findings but also contribute to a better understanding of these interconnected conditions. Overall, the study results demonstrate a rigorous approach to examining the quality of life and mental health of women with diabetes and cancer, offering valuable insights for both research and clinical practice.

## 5. Conclusions

This study aimed to investigate the psychosocial effects of comorbidity between T2DM and breast cancer in women. Our findings suggest that women with both conditions, particularly postmenopausal women over 60 years old, report a relatively higher mental well-being and QoL compared to those with T2DM alone. The relationship between these comorbidities and QoL is multifaceted, and our results point to the possibility that coping mechanisms, social support, or other unmeasured factors might contribute to the improved mental health outcomes in this unique group. Despite these positive findings, it is premature to conclude that the coexistence of T2DM and breast cancer does not affect QoL. Anxiety and depression remained prevalent even in the comorbid group, and the mechanisms driving the improvements in mental health and QoL observed require further investigation.

## Figures and Tables

**Table 1:** DASS-21 severity rating.

Severity	DASS-21-D	DASS-21-A	DASS-21-S
Normal	0–9	0–7	0–14
Mild	10–13	8–9	15–18
Moderate	14–20	10–14	19–25
Severe	21–27	15–19	26–33
Extremely severe	28+	20+	34+

**Table 2:** Demographic characteristics of all patients.

Demographic Characteristic		N.%
Age	31–45 46–60 61–85	10% 49.5% 40.5%
Family status	Married Single Widowed Divorced	49% 16% 27% 8%
Residence	Urban area Semi-urban area Rural area	58.9% 22.6% 18.5%
Education level	Primary Middle school High school Higher education Master’s/PhD	38.6% 18% 13.8% 22.6% 7%
Health insurance	Public Private Public and private No insurance	39.1% 40.4% 4.3% 16.2%
BMI	25 25–35 >35	25% 59.5% 15.5%
Occupation	Public worker Private worker Freelancer Retired Housekeeper Unemployed	16.5% 7% 5% 40.5% 19% 12%

**Table 3:** Clinical characteristics of all patients.

Clinical Characteristics		N.%		
		All	Τ2DM and Breast Cancer	Τ2DM
Chronic period of diabetes diagnosis	1–10 11–20 21+	55.1% 32.6% 12.3%	56.7% 36.7% 6.7%	53.6% 28.6% 17.9%
Therapeutic approach	Tablets Insulin Diet Tablets and insulin Diet and workouts	65.5% 18% 3% 11.5% 2%	87% 10% 3%	44% 26% 3% 23% 4%
Smoking?	Yes No	24.1% 75.9%	10% 90%	38.2% 61.8%
Alcohol?	Yes No	8.65% 91.35%	10% 90%	7.3% 92.7%
Complications of diabetes	Neuropathy Vasculopathy Heart attack Stroke Retinopathy Coronary artery disease Other complications	7.7% 9.8% 9.8% 5.5% 4.4% 4.4% 55.1%	6.6% 6.6% 6.6% 6.6% 6.6% 6.6% 66.7%	8.7% 13% 13% 4.3% 8.7% 8.7% 43.5%
HbA1c	<7 >7	76.7% 23.3%	79.2% 20.8%	74.1% 25.9%
Blood glucose	100–150 mg/dL 151–200 mg/dL 201–280 mg/dL	65.4% 24.4% 10.2%	65% 20% 15%	65.8% 28.9% 5.3%
Hypertension	Yes No	47% 53%	53% 47%	41% 59%
Menopausal state	Premenopausal Postmenopausal	26% 74%	13% 87%	39% 61%

**Table 4:** Correlation of demographic characteristics with the quality of life (according to ADDQoL analysis) in patients with T2DM and breast cancer from the two study groups.

ADDQoL with	Group A		Group B	
	Pearson’s r	*p*	Pearson’s r	*p*
Age	−0.083	0.743	0.303	0.034 *
Family status	0.190	0.375	0.057	0.673
Education	−0.126	0.566	−0.138	0.307
Residency	−0.078	0.716	0.254	0.061
Occupation	0.456	0.025 *	0.113	0.411
Health insurance	0.283	0.181	−0.284	0.051 *
BMI	0.208	0.330	0.159	0.275
Smoking	−0.020	0.917	0.096	0.475

* statistically significant *p*-values (*p* < 0.05).

**Table 5:** Correlation of clinical characteristics with quality of life (ADDQoL analysis) in the two study groups.

ADDQoL with	Group A		Group B	
	Pearson’s r	*p*	Pearson’s r	*p*
Time period with T2DM	0.061	0.758	0.193	0.154
Treatment for T2DM	0.176	0.361	0.094	0.483
Stage of cancer	−0.550	0.034 *	-	-
Treatment of cancer	0.245	0.124	-	-
Blood glucose	0.339	0.06	0.168	0.315
Hypertension	0.028	0.889	0.053	0.687
Menopausal state	0.096	0.620	0.288	0.035 *

* statistically significant *p*-values (*p* < 0.05).

**Table 6:** Comparisons of ADDQoL scores of women in both groups.

	Average ADDQoL Score		
	DM 2	DM2 and BC	*p*-Value
In general, my quality of life is…	0.69	0.23	0.037 *
If I did not have diabetes, my quality of life would be…	−1.22	−1.1	n/s
Domain			
1. Leisure activities	−2	−1.97	n/s
2. Working life	−3.17	−0.55	0.001 *
3. Local or long-distance journeys	−2.12	−1.31	0.07
4. Holidays	−2.05	−1.20	
5. Physical health	−2.38	−2.07	n/s
6. Family life/friendships	−2.40	−2.14	n/s
7. Social life	−1.57	−1.51	n/s
8. Close personal relationships	−1.22	−1.83	n/s
9. Sex life	−1.80	−0.83	0.05 *
10. Physical appearance	−1.72	−1.07	n/s
11. Self-confidence	−2.32	−1.80	n/s
12. Motivation to achieve things	−2.25	−1.72	n/s
13. People’s reactions	−1.67	−0.41	0.021 *
14. Feelings about the future	−2.83	−1.65	n/s
15. Financial situation	−1.38	−0.82	n/s
16. Living conditions	−1.87	−2.13	n/s
17. Dependence on others	−2.27	−2.31	n/s
18. Freedom to eat	−4.58	−2.86	0.002 *
19. Freedom to drink	−3.20	−3.03	n/s
Mean score of all questions	−2.21 (±0.84)	−1.64 (±0.7)	

* statistically significant *p*-values (*p* < 0.05).

**Table 7:** Correlation of demographic characteristics with anxiety and depression (according to DASS-21 analysis) in the two study groups.

DASS-21 with	Group A		Group B	
	Pearson’s r	*p*	Pearson’s r	*p*
Age	0.379	0.121	−0.086	0.556
Family status	−0.266	0.209	−0.047	0.730
Education	−0.100	0.605	−0.180	0.105
Residency	0.125	0.562	0.081	0.554
Occupation	−0.578	0.003 *	0.050	0.652
Health insurance	0.074	0.731	0.278	0.036 *
BMI	−0.122	0.507	0.046	0.751
Smoking	0.132	0.493	−0.107	0.435

* statistically significant *p*-values (*p* < 0.05).

**Table 8:** Correlation of clinical characteristics with anxiety and depression (according to DASS-21 analysis) in the two study groups.

DASS-21 with	Group A		Group B	
	Pearson’s r	*p*	Pearson’s r	*p*
Time period with T2DM	0.061	0.758	0.150	0.269
Treatment for T2DM	0.396	0.037 *	0.482	0.009 *
Stage of cancer	−0.550	0.034 *	-	-
Treatment of cancer	0.030	0.877	-	-
Blood glucose	0.339	0.06	0.268	0.104
Hypertension	0.028	0.889	−0.183	0.161
Menopausal state	0.096	0.620	−0.302	0.026

* statistically significant *p*-values (*p* < 0.05).

**Table 9:** Comparisons of women’s DASS-21 scores in both groups.

	Mean Score (DASS-21)					
	Depression			Anxiety		
Demographic Characteristic	T2DM	T2DM and Breast Ca	*p*-Value	T2DM	T2DM and Breast Ca	*p*-Value
Family status	Married: 18.12 Single: 21.66 Widowed: 20 Divorced: 14	13.57 13.33 14.36 ------	0.05 * 0.03 * n/s -	Married: 17.45 Single: 23 Widowed: 18.28 Divorced: 10	13.14 12.55 14 ----	0.05 * 0.02 * n/s -
Education level	Primary school: 24.28 Middle school: 17.14 High school: 24.22 Higher education: 18.18 Master’s/PhD: 9.5	15.6 13 8.8 - -	0.02 * n/s 0.001 * - -	Primary school: 24.4 Middle school: 16.5 High school: 19.09 Higher education: 14.7 Master’s/PhD: 16	14.7 12.5 6 - -	0.05 * n/s 0.003 * - -
Residence	Rural area: 22.57 Semi-urban area: 17 Urban area: 19.33	15.6 13.4 12.6	0.05 * n/s 0.03 *	Rural area: 21.6 Semi-urban area: 16.5 Urban area: 16.4	18.4 12.9 12.1	n/s n/s n/s
Occupation	Public worker: 16.6 Private worker: 19.5 Retired: 19.3 Unemployed: 14	4.6 - 1.23 24	0.001 * - 0.04 * 0.001 *	Public worker: 18.5 Private worker: 19.5 Retired: 15.8 Unemployed: 16.15	8 - 15.2 21.5	0.001 * - 0.05 * n/s
BMI	25: 18.8 25–35: 18.1 >35: 23.3	19.2 10.3 22	n/s 0.03 * n/s	25: 19.64 25–35: 16.42 >35: 22.6	19.7 10.1 16.6	n/s 0.01 * 0.05 *

* statistically significant *p*-values (*p* < 0.05).

**Table 10:** Comparisons of women’s DASS-21 scores in both groups.

DASS-21 Score		
T2DM	T2DM and Breast Ca	*p*-Value
Depression (D)		
15.7 (±12.2)	13.4 (±10)	0.378
Anxiety (A)		
16.2 (±11.6)	13.5 (±8.8)	0.281
Stress (S)		
17.3 (±8.2)	13 (±8.2)	0.049
Depression, Anxiety, and Stress (D + A + S)		

## Data Availability

The original contributions presented in this study are included in the article; further inquiries can be directed to the corresponding author.
